# The ‘difficult-to-treat depression’ and the ‘response paradigm’ models: Implications and relevance to patient management

**DOI:** 10.1177/00048674211013090

**Published:** 2021-05-10

**Authors:** RH McAllister-Williams, ST Aaronson, CR Conway, K Demyttenaere, PB Fitzgerald, CK Loo, PB Mitchell, AJ Rush, HA Sackeim, AH Young

**Affiliations:** 1 Northern Centre for Mood Disorders, Wolfson Research Centre, Campus for Ageing and Vitality, Newcastle University, Newcastle upon Tyne, UK; 2 Northumberland, Tyne and Wear NHS Foundation Trust, Newcastle upon Tyne, UK; 3 Department of Clinical Research, Sheppard Pratt Health System, Baltimore, MD, USA; 4 Department of Psychiatry, Washington University School of Medicine in St. Louis, St. Louis, MI, USA; 5 Faculty of Medicine, University Psychiatric Center, KU Leuven, Leuven, Belgium; 6 Epworth Healthcare, The Epworth Clinic, Melbourne, VIC, Australia; 7 Department of Psychiatry, Monash University, Melbourne, VIC, Australia; 8 School of Psychiatry, Faculty of Medicine, UNSW Sydney, Sydney, NSW, Australia; 9 Black Dog Institute, Sydney, NSW, Australia; 10 Duke University School of Medicine, Durham, NC, USA; 11 Texas Tech University Health Sciences Center, Midland, TX, USA; 12 Duke-NUS Medical School, Singapore; 13 Departments of Psychiatry and Radiology, Columbia University, New York, NY, USA; 14 Department of Psychological Medicine, South London and Maudsley NHS Foundation Trust, Institute of Psychiatry, Psychology & Neuroscience, King’s College London, London, UK

Royal Australian and New Zealand College of Psychiatry (RANZCP) guidelines have international impact. We read with enthusiasm the 2020 update of the mood disorders guidelines (Malhi et al., 2020a). There is much of value, certainly regarding medications. However, we found section 9 (‘Response to Treatment’, pp. 85–90) problematic in discussions of treatment-resistant depression (TRD) and the relatively new concept of ‘difficult-to-treat depression (DTD)’. The guidelines argue that ‘DTD is extremely heterogeneous, as any number and all manner of “difficulties” can contribute to non-response’ (p. 86). We agree, but do not see this as a weakness of the DTD model – rather a recognition of clinical reality of relevance to management. Of more concern, it is stated that ‘[DTD] does not sufficiently alter the focus of management’ (p. 86). We beg to differ.

Rather than TRD or DTD, adoption of a ‘response perspective’ model (proposed by Malhi et al., 2020b) is recommended (section 9.4, pp. 87–90). This model focuses on ‘response (outcome) and responsiveness (of the depression)’ (p. 87). While optimism about treatment is to be encouraged, the model appears to assert that virtually all patients with depression will eventually achieve sustained and substantial benefit from antidepressant treatment, and that the exceptions were wrongly diagnosed:

*the paradigm does allow for instances in which a specific treatment responsivity has not been found and all reasonable measures have been ineffective in achieving recovery. These are instances in which an alternative diagnosis is the likely cause of the depressive illness, for example, a stroke or neoplasm*. (p. 89)

While we endorse the need for further assessment and investigation of any patient who has not achieved recovery following multiple treatments, we believe that this statement, and the responsivity paradigm itself, ignores the clinical reality that such situations exist and are not simply related to some alternative diagnosis. Of note, remission rates beyond step 2 in the Sequenced Treatment Alternatives to Relieve Depression (STAR*D) study were less than 15% (Rush et al., 2006). This is precisely the point of the DTD model: it advocates regular review and re-assessment of treatment direction, acknowledging that in some situations the focus needs to shift from recovery to optimising symptom control and maximising psychosocial function (McAllister-Williams et al., 2020). The ‘response perspective’ ignores the prognostic importance of treatment history, clinical course and presentation in guiding treatment strategy. It sadly sidesteps the risk of a potentially endless sequence of treatment trials with ever-increasing side-effect burden while ignoring tractable reasons for poor outcomes.

Might the RANZCP guidelines be adjusted to address these concerns?
